# *In Vivo* Administration of a JAK3 Inhibitor to Chronically SIV Infected Rhesus Macaques Leads to NK Cell Depletion Associated with Transient Modest Increase in Viral Loads

**DOI:** 10.1371/journal.pone.0070992

**Published:** 2013-07-26

**Authors:** Yoshiaki Takahashi, Ann E. Mayne, Ladawan Khowawisetsut, Kovit Pattanapanyasat, Dawn Little, Francois Villinger, Aftab A. Ansari

**Affiliations:** 1 Department of Pathology and Laboratory Medicine, Emory University School of Medicine, Atlanta, Georgia, United States of America; 2 Department of Immunology, Faculty of Medicine Siriraj Hospital, Mahidol University, Bangkok, Thailand; 3 Office for Research and Development, Faculty of Medicine Siriraj Hospital, Mahidol University, Bangkok, Thailand; 4 Division of Pathology, Yerkes National Primate Research Center, Emory University, Atlanta, Georgia, United States of America; Rush University, United States of America

## Abstract

Innate immune responses are reasoned to play an important role during both acute and chronic SIV infection and play a deterministic role during the acute stages on the rate of infection and disease progression. NK cells are an integral part of the innate immune system but their role in influencing the course of SIV infection has been a subject of debate. As a means to delineate the effect of NK cells on SIV infection, use was made of a Janus kinase 3 (JAK3) inhibitor that has previously been shown to be effective in the depletion of NK cells *in vivo* in nonhuman primates (NHP). Extensive safety and *in vitro/in vivo* PK studies were conducted and an optimal dose that depletes NK cells and NK cell function *in vivo* identified. Six chronically SIV infected rhesus macaques, 3 with undetectable/low plasma viral loads and 3 with high plasma viral loads were administered a daily oral dose of 10 mg/kg for 35 days. Data obtained showed that, at the dose tested, the major cell lineage affected both in the blood and the GI tissues were the NK cells. Such depletion appeared to be associated with a transient increase in plasma and GI tissue viral loads. Whereas the number of NK cells returned to baseline values in the blood, the GI tissues remained depleted of NK cells for a prolonged period of time. Recent findings show that the JAK3 inhibitor utilized in the studies reported herein has a broader activity than previously reported with dose dependent effects on both JAK2 and JAK1 suggests that it is likely that multiple pathways are affected with the administration of this drug that needs to be taken into account. The findings reported herein are the first studies on the use of a JAK3 inhibitor in lentivirus infected NHP.

## Introduction

The fact that the net outcome of host-virus interactions during “acute” infection of both human HIV-1 infection and SIV infection of nonhuman primates dictates the rate of disease progression suggests that properties unique to the incoming virus and the quality and/or quantity of host “innate” immune effector mechanisms must play a deterministic role [1]. This view has led to the concept that it is during this time period post HIV/SIV infection that the die is already cast with regards to the rate of disease progression [2], [3]. While results of a recent study indicate properties such as replicative potential unique to the incoming virus [4] and/or differences in the anatomical tissue sites targeted by the virus [5] that appear to contribute to the rate of disease progression, results from a number of studies including our laboratory present an added and different perspective. Thus, studies utilizing single pools of stock SIV to infect groups of rhesus macaques showed a wide range of plasma and cellular viral loads at set point and diverse clinical outcome ranging from Elite Controllers to Fast Progressors [6]–[9]. These latter results suggest that while properties unique to the virus are important, the host innate and early adaptive immune effector mechanisms must play a dominant role during this acute infection period. However, the precise cell lineages that play this important role and the mechanisms by which innate and/or early adaptive immune effector cells mediate this important effect remains elusive.

One of the major cell lineage that comprise the innate immune effector mechanisms is the natural killer (NK) cells whose function in immune surveillance and mediating anti-viral effects have been recently reviewed [10], [11]. A large number of studies have characterized the development and differentiation of NK cells and its regulation [12]–[20] and documented both the phenotypic and functional heterogeneity that exists within the NK cell lineage [21]–[24]. Indeed, besides the classical non-MHC restricted cytolytic activity ascribed to NK cells, it is now being appreciated that there are subsets within this lineage that are non-cytolytic but can function to synthesize a variety of cytokines/chemokines [25], [26], serve to regulate immune function termed NKregs [27]–[32], serve as rheostats in controlling immune function [33] and most surprisingly acquire and maintain immunological memory [19], [34]–[36], although the mechanisms by which such “immunological memory” is manifested has been a subject of debate [37]. This finding of “immunological memory” along with the finding that NK cells have to undergo licensing and self MHC education [38]–[40], possess a degree of target antigen specificity [41] and display characteristics similar to T cells at the immunological synapse [42] continues to blur the previous demarcation between innate and adaptive immune function. These findings, thus, serve to make us re-assess our general view of NK cells as lacking specificity and as being evolutionary primitive and T cells having a high degree of antigen/MHC specificity and being more sophisticated [43], [44]. It is also important to recognize the fact that there are phenotypically and functionally distinct NK cells that are resident in specific organs and tissues such as the oral mucosa, gastro-intestinal tract (GIT) and the liver [22], [24], [41], [45], [46] that may be induced in these localized tissues to perform regular NK cell function in addition to unique functions such as tissue regeneration [47], [48] distinct from those conventionally ascribe to NK cells.

One of the most effective means to define the in vivo biological function of a given cell lineage is to devise methods for the selective and effective depletion of the cell lineage which for obvious ethical reasons can only be performed in experimental animals. Among the methods that have been employed so far for the depletion of NK cells include the administration of in vivo cell depleting lineage specific monoclonal/polyclonal antibodies, antibodies that neutralize growth factors/cytokines critically required for the growth or survival of the cell lineage and/or small molecule inhibitors of intracellular signaling pathways utilized primarily by this cell lineage [49]–[51]. In addition, the study of humans with a genetically inherited disease that results in NK cell depletion [52], [53], use of a transgenic murine strain deficient in NK cells [54], and the study of murine strains that show decline in NK cell function with age [55] exemplify the various lines of studies aimed at defining the in vivo role of NK cells. In the case of the use of antibodies to deplete the NK cell lineage, use has been made of an anti-asialo-GM1, anti-NK1.1 and anti-Ly49 antibodies in a variety of murine models [32], [49], [51], [56]–[58] with polarizing results in terms of beneficial versus detrimental clinical outcome depending on the model utilized [32]. The use of such antibodies to deplete NK cells, in addition, has important limitations as outlined elsewhere [59]–[61] that include the specificity of the cell lineage that is depleted. Germane to the studies reported herein, in the case of depleting NK cells in vivo in non-human primates, use has been made of an unique IL-2-diptheria toxin (denileukin) fusion protein [62], a murine anti-CD16 monoclonal antibody [63], [64], an antibody with specificity for IL-15 (required for NK cells) and a small molecule inhibitor with relative specificity for the JAK3 kinase an intra-cellular signaling molecule [65] to study the in vivo role of NK cells. Each of these reagents have varying levels of limitations and our laboratory thus chose to focus on optimizing the in vivo use of a small molecule relatively selective JAK3/1 inhibitor [66] termed CP-690,550 whose activity has recently been compared with other JAK inhibitors [67] and while initially thought to be specific for the inhibition of JAK3, has more recently shown to also inhibit JAK1/2. We chose to utilize CP-690,550 because the drug targets the JAK-STAT pathway utilized for NK cell activation and JAK3, which along with JAK1 relays cytokine signaling through the cytokine receptor common gamma chain also known as CD132. The JAK/STAT pathway is activated not only in NK cells but other cell lineages that in concert are involved in the innate and early immune responses [68], [69]. In addition, its plasma in vivo levels could be easily monitored allowing for a more objective pharmacokinetic analysis and its use in nonhuman primates has previously shown significant in vivo activity against NK cells [65] with limited effects on CD8^+^ T cell numbers, and no effect on effector CD8^+^ T cell function [70]

To initiate studies for the inhibition of innate and early immune responses using CP-690,550 (heretofore referred to as the JAK3 inhibitor) in the rhesus macaque species, a detailed in vitro specificity and a series of in vivo safety and pharmacokinetic study was first conducted and based on the results of the PK studies an optimal dose was selected and utilized to define the effect of this novel JAK3 inhibitor on influencing the course of disease in a group of chronically SIV infected rhesus macaques. Results of these studies constitute the basis of this report.

## Materials and Methods

### Animals and Source of Blood & Colo-rectal biopsy Samples

Juvenile to adult male rhesus macaques (Macaca mulatta) of Indian origin were used for the studies reported herein. The animals were all housed at the Yerkes National Primate Research Center (YNPRC) of Emory University (Atlanta, GA) and were maintained according to the guidelines of the Committee on the Care and Use of Laboratory Animals of the Institute of Laboratory Animal Resources, National Research Council and the Department of Health and Human Service guideline titled Guide for the Care and Use of Laboratory Animals. The initial in vitro and in vivo safety and pharmacokinetic studies were performed utilizing a group of 9 healthy rhesus macaques that initially served as normal blood donors and then were utilized for a number of pharmacokinetic studies outlined below. The subsequent in vivo studies of CP-690,550 were conducted on a group of 6 chronically SIV infected adult male rhesus macaques and for purposes of control 12 similarly SIV infected rhesus macaques. Each monkey was infected intravenously with 1000 TCID_50_ of SIVmac239 (grown in day 3 Con-A activated normal rhesus PBMC cultures). Viral loads were monitored in plasma samples every week for 4 weeks, every other week for 8 weeks and monthly thereafter, using bDNA quantitation on aliquots of EDTA plasma by Siemens Inc. (Berkeley, CA). Cellular viral loads in aliquots of PBMC and colo-rectal biopsy tissue samples were monitored using a technique previously outlined [71]. Blood and colo-rectal biopsy samples were obtained prior to and at 1, 3, 6, 8 and 12 weeks after initiation of the JAK inhibitor.

### Ethics Statement

All animals were born and maintained at the Yerkes National Primate Research Center of Emory University in accordance with the regulations of the Committee on the Care and Use of Laboratory Animal Resources. The animals are fed monkey diet (Purina) supplemented daily with fresh fruit or vegetables and water ad libitum. Additional enrichment including the delivery of appropriate safe toys is provided and overseen by the Yerkes enrichment staff and animal health is monitored daily by the animal care staff and veterinary personnel, available 24/7. Monkeys are caged in socially compatible same sex pairs to facilitate social enhancement and well-being. Monkeys showing signs of sustained weight loss, disease or distress are subject to clinical diagnosis based on symptoms and then provided either standard dietary supplementation analgesics and/or chemotherapy. Monkeys whose symptoms cannot be alleviated using standard dietary supplementation, analgesics and/or chemotherapy were humanely euthanized using an overdose of barbiturates according to the guidelines of the American Veterinary Medical Association. The studies reported herein were performed under IACUC protocol #2001186 “Innate immunity in SIV infection” which was reviewed and approved by the Emory University IACUC. It has been assigned the IACUC protocol number “YER-2001186-082414GA”. The Yerkes National Primate Research Center has been fully accredited by the Association for Assessment and Accreditation of Laboratory Animal Care International since 1985. All experiments were reviewed and approved by the Emory institutional animal use and care as well as biosafety review Committees.

### CP-690,550

This JAK-3 inhibitor was either synthesized by the Chemistry Lab at Emory University or purchased from LC Laboratories (Woburn, MA) and an aliquot (10 mg) was a kind gift from Pfizer Labs, Inc. (Groton, CT). The aliquot from Pfizer was utilized for quality comparison of the other lots of the same compound. For in vitro studies, a stock solution of the compound CP-690,550 (313 m.w.) was prepared by first dissolving it in DMSO and then in methanol to obtain a 2 mM solution and then further diluted to the required concentration in sterile culture medium. For the dosing of monkeys, the compound in 0.5% methylcellulose was incorporated in peanut butter and administered to monkeys orally based at a loading dose of 20 mg/kg and then subsequently at 10 mg/kg daily for a period of 35 days except for the initial PK studies that were conducted utilizing doses as described in the text. Quantitation of plasma levels of the CP-690,550 was performed using a reverse phase-HPLC with MS/MS with a detection level sensitivity of 2.5 ng/ml [72]. Safety of the drug was determined by the analysis of blood samples from the monkeys administered 30 mg/kg (highest dose) by performing complete hematologic, metabolic, renal, liver and cardiac function tests and found to have no detectable toxicity. All data have been recorded and on file for purposes of reference.

### *p-STAT-5 Assay

The signal transducers and activators of transcription (STATs) are a family of intracellular molecules consisting of seven transcription factors that respond to a variety of stimuli including cytokines, hormones, and growth factors [73]. Phosphorylation of the tyrosine residues of the STAT molecules leads to their activation which following dimerization translocates to the nucleus and contributes to the activation of appropriate target genes. Janus kinases (JAKs) are the major source that leads to STAT phosphorylation and this JAK/STAT pathway plays a critical role in communicating cell surface dialogue into immune function (Harrison DA). In the studies reported herein we studied the phosphorylation of STAT5, which is mediated by several JAKs, including JAK3 to monitor the activity of the JAK3 inhibitor. Standard Western Blots of cell lysates were performed according to the manufacturers instruction and with appropriate positive and negative controls. The kit was purchased from Zeptometrix Corp, Buffalo, NY.

### *In vitro* NK cell assay

The JAM assay [74] was utilized to assay for the evaluation of NK cell function in PBMC samples from rhesus macaques as our laboratory has described elsewhere [23]. The MHC class I negative human erythro-leukemic K562 cell line was used as a target in these studies. The K562 cell line was obtained from ATCC, Rockville, MD. Effector cell: target cell ratios of 50, 25, 12.5 and 6.25∶1 in triplicate were used in each assay. Data are expressed as lytic units (L.U.) per 10^7^ effector cells as describe elsewhere [23]. The S.D. of L.U. by the effector cells was <10% for all the assays performed. The in vitro assay for the effect on NK cell function was also carried out in the presence of 50 mg/ml of human albumin to test the potential role of serum components and found to have minimal (<5%) effect on the NK cell functional assay.

### Flow cytometric analyses

Aliquots of ficoll-hypaque gradient purified peripheral blood mononuclear cells (PBMC) and mononuclear cells isolated from a pool of colo-rectal biopsies according to techniques previously described [71] were utilized to determine the frequencies of varying subsets of lymphoid cells. CBC values were used to determine the absolute numbers of each subset. A battery of monoclonal antibody reagents that react with antigens expressed by cells from rhesus macaques conjugated with a variety of fluorochromes was used for polychromatic flow cytometric analysis utilizing a LSR-II flow cytometer (B–D Immunocytometry Division, Mountain View, CA). The ﬂuorochrome-conjugated monoclonal antibodies (mAbs) were used in appropriate combinations to stain the mononuclear cells to determine the frequencies of cell lineages isolated from the blood and rectal biopsies with a focus on T cell, NK cell and dendritic cell lineages. The mAbs purchased from BD Biosciences (San Jose, CA) included Alexa700-anti-CD3 (clone SP34-2), FITC-anti-CD8 (clone SK1 and clone RPA-T8), PerCP or Pac Blue anti-CD4 (clone L200), FITC-anti-CD14 (clone M5E2), PerCP-Cy5.5-anti-CD16 (clone 3G8), APC-Cy7-anti-CD20 (clone 2H7), and PE-Cy7-anti-CD56 (clone NCAM16.2). The mAb purchased from Beckman Coulter (Brea, CA) included PE-anti-NKG2a (clone Z199), the mAb Alexa647-anti-IL-17A (clone eBio64DEC17) was purchased from eBioscience (San Diego, CA) and the mAb APC-anti-IFN-α (clone LT27:295) and PE/APC-anti-CD25 (clone 4E3) was purchased from Miltenyi (Auburn, CA). Select studies utilized anti-Ki-67 and PE-conjugated p11c-Mamu-A01*-MHC class I tetramer (courtesy The NIH tetramer core facility, Emory University School of Medicine, Atlanta, GA). A minimum of 40,000 events was analyzed and the FlowJo (Treestar, Ashland, OR) software utilized for data analysis. Phenotypically NK cells were defined as cells that were CD3^−^, CD8^+^, NKG2a^+^ which includes the 4 major subsets of NK cells as described elsewhere [23].

### Statistical analysis

All statistical including the graphical analysis was performed using the SPSS software (SPSS Inc., Chicago, IL). The non-parametric Mann-Whitney U test was used to compare the data sets between groups of monkeys. The differences with a p-value less than 0.05 were considered as statistically significant.

## Results

Studies were first carried out to establish the pharmacokinetics and safety of the oral administration of the JAK3 inhibitor in 2 adult rhesus macaques. The monkeys received a single oral dose of 10, 20 and 30 mg/kg of the JAK inhibitor after a week long wash out period in between. Results of these studies showed no evidence of hematologic, liver, renal and/or metabolic toxicity at the doses administered. Mean plasma levels of the JAK inhibitor peaked at 2 hours and the trough levels appeared at about 6 hours post administration. It was determined that dose levels of 10, 20 and 30 mg/kg resulted in trough mean plasma concentrations of 3.14+/−0.08, 5.67+/−1.4 and 9.44+/−3.27 ng/ml, respectively. Based on the results of phenotypic analyses and the NK cell functional studies (see below) it was decided that a loading dose of 20 mg/kg followed by a daily dose of 10 mg/kg in adult monkeys would provide appropriate trough levels to deplete NK cells and was the dose to utilize and hence this dose level was utilized for all subsequent studies.

### *In vitro* and *in vivo* effect of the JAK3 inhibitor on NK cell activity

**Figure 1:**
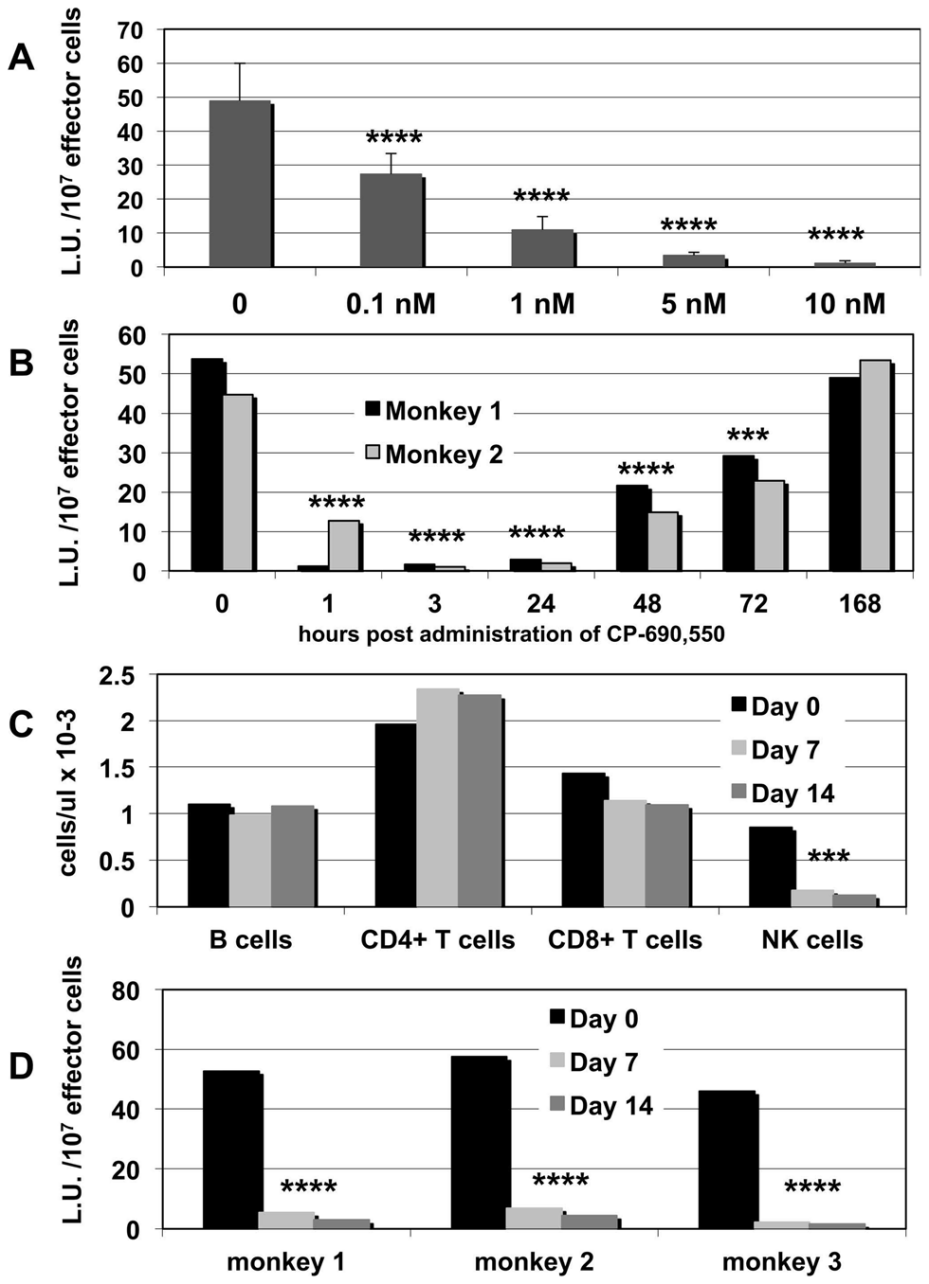
The effect of the JAK3 inhibitor on rhesus macaque NK cell function in vitro/ in vivo and on the frequencies of the major cell lineages in the blood. A) PBMC from 9 normal rhesus macaques was assayed for NK activity in the presence and absence of varying concentrations of the JAK-3 inhibitor. The mean lytic units/10^6^ effector cells were calculated. The S.D. was always <10%. B) Two normal rhesus macaques were orally administered 10 mg/kg of the JAK3 inhibitor and PBMC samples obtained prior to and at 1, 3, 24, 48, 72 and 168 hrs. post JAK3 administration and assayed for NK activity. Results reflect Mean lytic units of NK activity/10^6^ effector cells performed in triplicated with the S.D. <10%. C) Three normal rhesus macaques were orally administered the JAK3 inhibitor at 10 mg/kg daily for 14 days and an aliquot of their PBMC obtained prior to and at day 7 and 14 subjected to polychromatic flow cytometric analysis for the frequencies of CD4^+^ T cells, CD8^+^ T cells, CD20^+^ B cells and CD3^−^/CD8^+^/NKG2a^+^ cells. An aliquot was utilized for CBC and the absolute numbers of each cell lineage calculated and illustrated. Note the major depletion of the NK cells D). Another aliquot of the PBMC from C) were assayed for NK cell function and the data shows the Mean lytic units/10^6^ effector cells. The S.D. was always <10%.

PBMC from a total of 9 rhesus macaques were assayed for NK cell activity in vitro using our standard NK cell functional assay. As seen in Fig. 1A, as little as 0.1 nM of the JAK3 inhibitor significantly (p<0.0001) reduced NK cell function showing maximal inhibition at 5–10 nM. The calculated IC_50_ was shown to be at 1nM for NK cell inhibition. In vivo functional PK studies were performed next. Two adult rhesus macaques were orally administered 10 mg/kg of the JAK3 inhibitor and blood samples obtained at various time intervals and assayed for NK cell activity. As seen in Fig. 1B significant inhibition (p<0.0001) of NK cell function was seen as early as 1 hour and as late as 72 h following a single dose of 10 mg/kg of the JAK3 inhibitor. These studies were followed by dosing of 3 rhesus macaques with a daily oral dose of 10 mg/Kg for 14 days and both the frequencies of CD4^+^T, CD8^+^T, B and NK cells and the functional NK cell activity of blood samples obtained at day 0 (baseline) and days 7 and 14 were conducted. Results of these studies clearly showed marked selective depletion of NK cells associated with a sharp reduction (p<0.0001) of NK cell function (Fig. 1C & D). There was also a modest decrease in the numbers of the gated population of CD3^+^ CD8^+^ T cells that could reflect a subset of NK cells that express these markers. Aliquots of the PBMC samples incubated in vitro with the JAK3 inhibitor were also monitored for the phosphorylation of STAT-5 (*p-STAT-5) as described in the methods section and found to show compete inhibition (data not shown). What was also interesting from the data obtained was the finding that there did not appear to be any selective ordered re-appearance of NK cell subsets post depletion during the period of reconstitution. Thus, as we have established before, there are at least 4 subsets of NK cells in rhesus macaques [23]. These include the CD3^−^, CD8^+^, NKG2a^+^ that are CD16^+^, CD56^−^ (the major cytolytic subset), those that are CD16^−^, CD56^+^ (cytokine synthesizing) and the CD16^−^, CD56^−^ and the CD16^+^, CD56^+^ subsets. In addition, there are also the NK-17 cells and the NKregs [6]. Careful detailed polychromatic flow cytometric analysis of PBMC's for these subsets of NK cells from all 6 monkeys administered the JAK3 inhibitor failed to show any specific pattern of subset evolvement at least in the PBMC samples. Thus, at least phenotypically, we were unable to show the emergence of one particular NK cell subset over another in order to make the case for a correlation between cell surface markers and progenitor to mature cell development using PBMC samples. The reasons for this are unclear at present.

### Effect of the *in vivo* administration of the JAK3 inhibitor on cell subsets and viral loads in SIV infected rhesus macaques

The primary objective of this study was to determine the in vivo role of the JAK3 inhibitor, if any, on influencing viral loads in monkeys chronically infected with SIV. Three monkeys were selected that were classified as spontaneous controllers with one showing low levels and 2/3 showing undetectable plasma viremia and 3 additional monkeys that displayed relatively higher viral loads (>100,000 viral copies/ml of plasma) in efforts to determine if differences in viral loads influenced the outcome of the administration of the JAK3 inhibitor. Plasma specimens were monitored for viral loads prior to and weekly intervals for 4 weeks, bi-weekly until 14 weeks post administration of the JAK3 inhibitor. PBMC's and colo-rectal biopsies were monitored for the frequency of mononuclear cell subsets by standard flow cytometry, cellular viral loads prior to, at 1, 3, 6, 8 and 12 weeks post JAK3 administration and for NK function. An aliquot of the PBMC was also monitored for *p-STAT-5.

**Figure 2:**
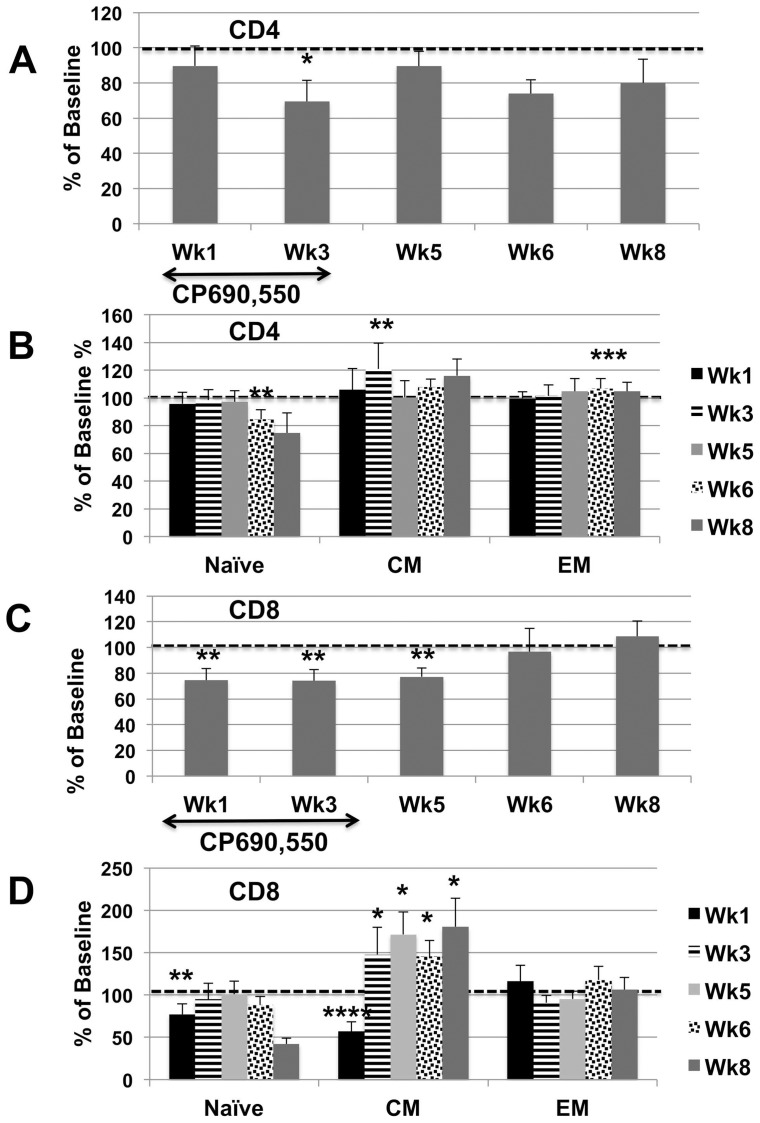
The effect of JAK3 oral administration on the frequencies of CD4^+^ and CD8^+^ T cells and their subsets in 6 SIV chronically infected rhesus macaques. Each animal received an initial loading does of 20 mg/kg and thereafter a daily dose of 10 mg/kg for a total of 35 days. A) Aliquots of the PBMC obtained prior to (baseline) and at weeks 1, 3, 5, 6, 8 and 12 were analyzed for the frequencies of lymphoid cell subsets A) The mean % change from baseline values of CD4^+^ T cells is shown. B) The mean % change from baseline values of CD4^+^ T cells classified as naïve, central memory (CM) and effector memory (EM) is illustrated c) The mean % change from baseline of total CD8^+^ T cells is shown and D) The mean % change from baseline values of CD8^+^ T cells classified as naïve, central memory (CM) and effector memory (EM) is illustrated. Statistical differences are denote as asterisks with * (p<0.01), ** (p<0.05), *** (p<0.03) and **** (p<0.001).

**Figure 3:**
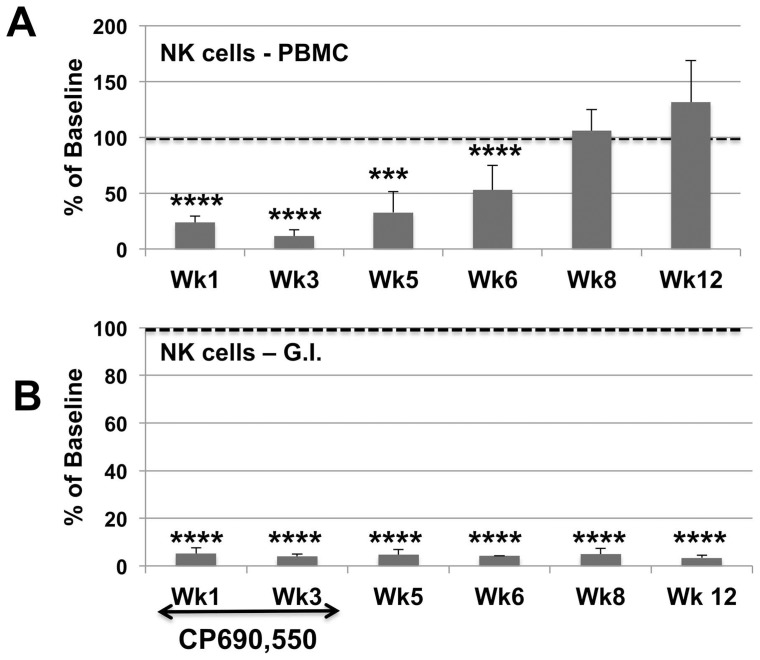
Frequencies of CD3^-^/CD8^+^/NKG2a^+^ (NK cells). Aliquots of cells from the same 6 monkeys as described under Fig. 2 were assayed for the frequencies of CD3^−^/CD8^+^/NKG2a^+^ (NK cells) and the mean % change from baseline values illustrated for A) PBMC samples and B) Gastro-intestinal tissue samples. There was high statistical difference in the values obtained on both PBMC samples (p<0.001) and G.I. tissue samples (all p<0.0) that did not return to baseline values.

**Figure 4:**
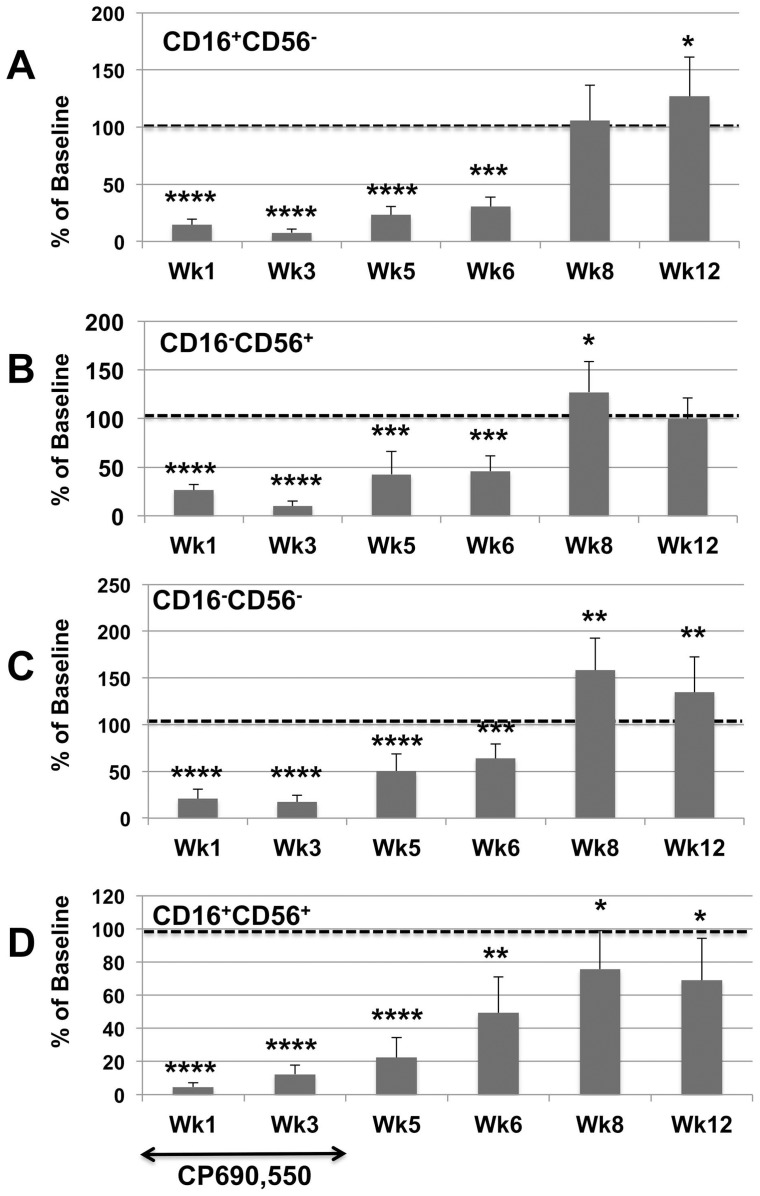
Frequencies of the 4 major subset of the CD3^−^/CD8^+^/NKG2a^+^ NK cells. Aliquots of the same PBMC from the 6 SIV infected monkeys as used under Fig. 2 were also analyzed for the frequencies of the 4 major subset of the CD3^−^/CD8^+^/NKG2a^+^ NK cells. The data is once again expressed as the mean +/− S.D. of the baseline values from individual monkeys. A) The % change from baseline values for the CD16^+^/CD56^−^ (cytolytic subset) B) The % change from baseline for the CD16^−^/CD56^+^ (major cytokine synthesizing subset) C) The % change from baseline in the CD16-/CD56- subset and D) The % change from baseline of the CD16^+^/CD56^+^ subset. The statistical differences are denoted by asterisks and reflects * (p<0.01), ** (p<0.05), *** (p<0.03) and **** (p<0.0001).

Due to marked differences in plasma viral loads accompanied by marked variations in the absolute number of various lymphoid cell subsets at baseline in the individual animals being studied, the data on phenotypic analysis of subsets of PBMC are illustrated to a large extent as a percent of baseline values. For purposes of reference, the absolute numbers of each major subset at baseline is illustrated in Supplemental Fig. S1. As seen in Fig. 2A, the administration of the JAK3 inhibitor at the doses specified led to a decrease (p<0.01) in the frequencies of CD4^+^ T cells in the PBMC with a nadir at week 3, but with no detectable net change in the frequencies of CD4^+^ T cells in the colo-rectal tissues (data not shown). Analysis of subsets of CD4^+^ T cells in these samples showed a decrease (p<0.05) in the relative frequencies of CD4^+^ naïve (CD28^+^, CD95^−^, CCR7^+^) at week 6 and 8 and an increase (p<0.05) in the central memory (CM) CD4^+^ T cells (CD28^+^, CD95^+^, CCR7^+^) at week one and an increase (p<0.03) in the effector memory (EM) CD4^+^ T cells (CD28^−^, CD95^+^, CCR7^−^) at week 6 (Fig. 2B). There was also a significant decrease in the frequencies of CD8^+^ T cells at weeks 1, 3 and 5 (p<0.05) but was followed soon thereafter with a return to baseline values (Fig. 2C). Major findings of the subset analysis of CD8^+^ T cells showed a marked decrease in the frequencies of CM CD8^+^ T cells at one week (p<0.001) but followed by marked sustained increase (p<0.01) thereafter (Fig. 2D). Of interest there were no statistical difference in the numbers of CD3^+^, CD8^+^ T cells in the corresponding gut tissues. A study of these same subsets in a cohort of 12 similarly chronically SIV infected rhesus macaques studied in parallel basically showed a gradual decrease in CD4^+^ T cells with an increase in the frequencies of CD8^+^ T cells but the differences were not as striking as those outlined above following JAK3 inhibitor administration (data not shown for purposes of brevity). However, of all the changes noted with the administration of the JAK3 inhibitor, the most marked change (p<0.0001) noted was in the number of CD3^−^, CD8^+^, NKG2a^+^ (NK cells) both in the PBMC and the colo-rectal biopsy tissues (see Fig. 3 A and B). Thus there appeared to be between 80–90% depletion of the NK cells from both the blood and gut tissues starting at one week of initiation of JAK3 inhibitor administration and sustained until 6 weeks following cessation of drug therapy in the PBMC. It is of interest to note, however, that, whereas the numbers of NK cells gradually started returning to baseline values in the blood, the values of NK cells in the colo-rectal tissues remained low until week 12 and up to the time period these animals were followed (>36 weeks). The frequencies of the IL-17 synthesizing NK cells (NK-17) were also monitored as previously described [6]. The frequencies of the NK-17 cells were reduced to undetectable levels in both the PBMC and colo-rectal biopsy tissues. Data on analysis of the 4 major subsets of NK cells basically mirrored the profile seen with the total NKG2a^+^ population (Fig. 4 A, B, C and D) with marked decreases at week 1, 3, and 5 (p<0.0001) to a gradual return to baseline values at weeks 6 and in most cases an increase at week 8. Functional studies on NK cell activity was also monitored and found to be below the level of detection in our standard NK cell assay for samples from 1 to 6 weeks that returned to normal pre-baseline levels thereafter (data not shown).

**Figure 5:**
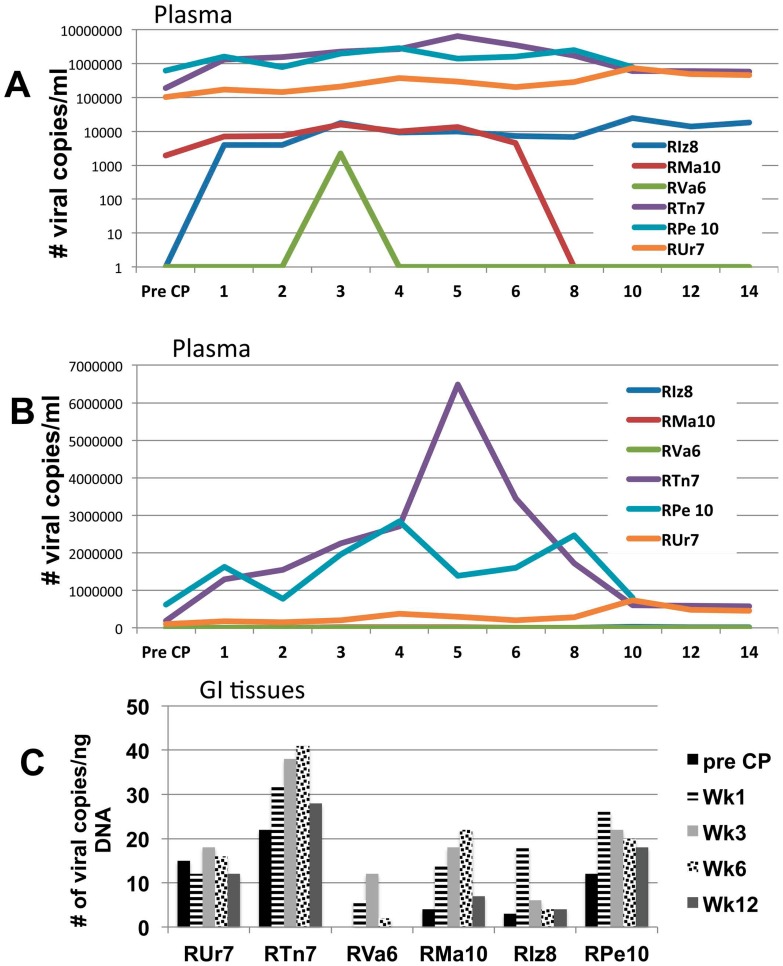
Effect of JAK3 inhibitor on plasma and gastro-intestinal tissue viral loads. Aliquots of the plasma samples obtained prior to and post administration of the JAK3 inhibitor from each of the 6 monkeys were assayed for levels of SIV (commercially performed). A) Reflects # of viral copies/ml of plasma obtained from each of the 6 monkeys at weekly intervals until week 6, bi-weekly until week 14 is illustrated using a log scale to highlight differences seen in monkeys with low viral loads B) Reflects # of viral copies/ml of plasma obtained from each of the 6 monkeys at weekly intervals until week 6, bi-weekly until week 14 is illustrated using an arithmetic scale to highlight differences seen in monkeys with high viral loads C) Reflects the viral copies/ng DNA isolated from a pool of colo-rectal biopsies from each of the 6 monkeys obtained prior to (baseline) and at weeks 1, 3, 6 and 12 post JAK3 inhibitor administration.

Plasma viral loads and colo-rectal biopsy tissue pro-viral loads were monitored in these same monkeys in efforts to determine the effect of JAK3 inhibitor administration. It should be noted, we had purposely selected 3 monkeys that had high viral loads (HVL) and 3 with low to undetectable levels of viremia to determine if the level of viral loads at the initiation of JAK3 inhibitor administration would play a role. As seen in Fig. 5 A & B (both log and arithmetic scale viral loads are shown due to the differences in the levels in the 2 subset of monkeys), 2/3 monkeys in each of the 2 subset show a modest but significant increase in plasma viral loads at about 1–2 weeks of JAK3 inhibitor administration which gradually returned to pre-JAK3 inhibitor administration levels shortly following cessation of drug administration. The levels of pro-viral DNA in the gut tissues also increased to different levels in all 6 animals (Fig. 5 C), with the 3 monkeys with lower plasma VL, RVa, RIz and RMa having lower cellular VL than the other 3 with higher plasma VL. Five of the 6 monkeys clearly also showed a marked increase in gut tissue VL (Fig. 5C) denoting that treatment with the JAK3 inhibitor had effects on gut tissue VL. The fold increase although transient appeared of interest to be more significant in the 3 animals with low to undetectable viremia (5–6 fold) as compared with the 3 animals that had high viral loads (approx. 2 fold). Analysis of the colo-rectal biopsy tissues for the frequencies of CD4^+^ T cells, as mentioned above, failed to show any marked differences from previous baseline levels suggesting that the increases noted was not due to increased frequencies of CD4^+^ T cells but likely due to increased viral output per infected cell.

## Discussion

The results of the studies reported herein show that this JAK inhibitor clearly has potent inhibitory effect on NK cells in vitro and that the in vivo administration of this inhibitor which was previously thought to be specific for JAK3 [75]–[77] but has recently been shown to have a broader JAK species activity [67], [78], based on the doses tested, did indeed result in marked depletion of the NK cell lineage with relatively smaller depletion of the other cell lineages. Such depletion appears to result in modest but detectable increases in plasma and gut tissue viral loads in all rhesus macaques chronically infected with SIV. It is of interest to note that these increases were seen in monkeys with both low to undetectable viral loads and also in monkeys with significantly high viral loads suggesting that the drug and/or depletion of the NK cells induces the neo-activation of cryptically infected target cells but could also lead to higher viral load output from the cells previously replicating virus. A number of issues from these studies need address. First of all, are the increases in plasma and gut tissue viral loads really significant and/or meaningful? What is the specificity of this JAK inhibitor and specially its effect on other cell lineages? What are the reasons for the lack of gut tissue NK cell replacement and its implications? What cell lineage(s) is the source of the emergence of viremia following drug administration in the animals with low to undetectable viremia? Does this depletion of NK cells lead to any changes in the adaptive immune response of the animals? Attempts were made to address each of these issues.

The first issue concerns the significance of the changes in plasma and gut tissue viral loads. While statistical analysis of the data failed to show significance, it is clear that the trend for increased viral load was evident for all 6 animals. It appears that a much larger cohort of animals needs to be studied to derive statistically valid data. Of note are the levels of differences in the 2 sets of animals with differences in baseline plasma viral loads. Thus, 2/3 animals with undetectable plasma viral loads showed a peak increase to 2261 and 25635 viral copies/ml and the 3^rd^ animal showed an increase from 1962 to a peak level of 16139 viral copies/ml. Of the monkeys with higher baseline plasma viral loads, the peak increases noted were from 618,967 to 2,984,174 copies/ml, from 101,380 to 740,217 copies/ml and 188,972 to 6,591,000 copies/ml. The fold increases in GI tissue viral loads paralleled that noted in the plasma. Thus, 2/3 with undetectable viral loads increased to peak levels of 18 and 12 copies/ng DNA and the 3^rd^ increased from 4 copies to peak levels of 22 copies/ng DNA. The monkeys with higher baseline GI tissues viral loads increased from 12, 15 and 22 copies/ng DNA to peak levels of 26, 18 and 41 copies/ng DNA, respectively. Two issues seem obvious. Firstly, the fact that both the plasma and GI tissue viral loads appear to return to baseline levels indicates that some regulatory mechanisms maybe operable in these animals. Phenotypic analyses appear to indicate that the rapid return of NK cells directly correlates with the reduction of plasma and gut tissue viral loads. While one can argue whether this is truly due to the return of the NK cell lineage, results from a study performed by Dr. M. C. Gauduin seems to support that this is likely due to NK cells (personal communication). Thus, the study involved the depletion of CD8^+^ cells in a group of chronically SIV infected rhesus macaques following treatment with ART. The question being addressed was whether CD8^+^ cells were regulating viral reservoirs. The plasma viral loads rapidly increased in these animals but returned to set point levels but this decrease to set point levels was associated with the return of classic CD16^+^, CD56^−^, NKG2a^+^ NK cells [79] but not either CD3^+^ CD8^+^ T cells and/or the frequencies of Mamu-A01-p11C tetramer^+^ cells. These data suggest that the NK cells are likely the cells initially involved in decreases of viral loads. The second issues with regards to the studies reported herein concerns whether the increases in viral load is due to more viral output from cells already producing virus or is it due to the neo-activation of latently infected cells? We are currently attempting to address both these issue in a separate study of SIV infected Elite Controller rhesus macaques.

With regards to the specificity of the JAK3 inhibitor, while these studies were initiated at a time when the published data supported the view that the drug had high specificity for JAK3, it is becoming increasingly apparent that the drug inhibits JAK1 and to some degree also JAK2. Some discussion on the JAK/STAT pathway thus appears appropriate. There are some excellent reviews on this subject [68], [80] and thus will only be summarized here. The Janus kinases are a family of 4 enzymes JAK1, JAK2, JAK3 and tyrosine kinase 2 (TYK2) that are integrally involved in intracellular signaling. Each JAK family enzyme is involved and inter-connected with individual cytokine receptor mediated signaling cascades through the corresponding enzymes of signal transducers and activators of transcription (STAT) family. JAK3 acts in concert with JAK1 to transmit signaling via the cytokine receptor common gamma chain. JAK2 is important in signaling mediated by hormones and growth factors such as erythropoietin and GM-CSF via the IL-3R beta chain. JAK1, JAK2 and TYK2 are involved in IL-6 signaling. The JAK-STAT induced signaling events are in turn negatively regulated by the suppressors of cytokine signaling (SOCS). What is interesting is that each of these enzymes appear to be activated by specific cytokines which makes the identification of inhibitors specific for each enzyme translate into the targeting of specific diseases. This is precisely the reason for an enormous interest by the pharmaceutical industry on identifying inhibitors of specific JAK/STAT enzymes [78]. The commercial name for CP-690,550 is Tofacitinib and the trade name is Xeljanz and this drug has shown promising results in clinical trials for the therapy of rheumatoid arthritis [81], [82]. Given the inter-connectivity between the various JAK's, it is thus not surprising that the administration of the JAK3 inhibitor affected several cell lineages. Based on the data reported herein, however, it seems that there does appear to be an effect of dose on the scope of cell lineages that become affected. Clearly, the most affected cell population at the doses utilized herein appeared to be the NK cell lineage. This could be due to the density of the high affinity IL-2R expressed by the NK cells. Nonetheless, it is clear that the data obtained with the use of this JAK3 inhibitor has to be interpreted with caution.

Attempts were made to draw parallels between NK cell subsets depletion/re-emergence in the SIV infected monkeys with observations of NK cell subsets in humans. Thus, it is clear that there are at least 2 major phenotypically defined subsets in human. Those that express CD56^low^/CD16^+^, a major cytolytic subset and those that express CD56^bright^/CD16^low^, the major cytokine synthesizing subset [83]. The major CD56^low^/CD16^+^ subset is also heterogeneous and composed of cells that are CD122^+^/CCR7^−^ and CD122^−^/CCR7^+^. The former subset (CD122+/CCR7−) has been shown to be expanded following HIV-1 infection that also express the senescence marker CD57 [84]and is likely the same subset that shows increased KIR expression [85]. As noted above, the finding of >90% depletion of the NK cell lineage in the PBMC and GI tissues prompted us to determine if the re-emergence rate of the various NK cell subset will provide clues as to the pattern of differentiation of this cell lineage as outlined by our lab previously [86]. Unfortunately, no clear pattern emerged, as it appeared that each of the 4 major subsets including the human equivalent of the cytolytic and cytokine producing subset appeared with a tempo that reflected their individual frequencies prior to depletion. The reason for this finding is not clear at present and may be because we only sampled the blood/GI tissues and not the bone marrow and the sampling of the GI tissue was conducted between large intervals of time. In this regard, it is also of interest to us that once depleted in the GI tissue, this cell lineage did not reconstitute in this tissue location but appeared to be replaced by fibrosis (data not shown). This finding was surprising and is being currently studied. Preliminary data seem to indicate that the JAK3 inhibitor markedly inhibits the expression of the gut homing molecules α4β7 and CCR9 by NK cells in vitro. However, this does not explain the failure of NK cells to express such gut homing molecules at a time period when the presence of the drug in vivo is highly unlikely. Studies of KIR expression by these NK cells prior to and following SIV infection has also been studied by our laboratory and reported elsewhere [87].

With regards to the source of the modest increase in viremia, it is clear that the source of the virus in monkeys that had undetectable plasma viremia at baseline was from CD4^+^ T cells as determined by in situ hybridization analysis. However, it is not clear as the source of virus in animals, which had high plasma viremia. This question while simple at face value is in fact a very difficult issue to address since there is currently no acceptable assay to measure burst size for SIV. This question has important implication in the field of HIV/SIV research. Thus we do not know if changes in viremia are due to increased output from a cell or due to increase in the number of cells that are secreting virus. Even if this is addressed, the issue as to whether the virus being measured is dead or alive (since we utilize PCR) and/or is replication competent or not remains an open question.

We did attempt to determine whether depletion of NK cells had any effect on adaptive immune responses in these monkeys. Thus, we measured levels of anti-SIV antibodies by ELISA and performed ELISPOT assays to determine whether differences could be detected. Unfortunately, we were unable to detect any reproducible difference in either the levels of anti-SIV antibody and/or the number of ELISPOT values when compared to either baseline values and/or values obtained on the control SIV infected monkeys assayed in parallel. This is not surprising since a role for NK cells on modulating adaptive immune responses during chronic infection is likely to be limited. It should be noted that monitoring of the appearance of Ki67 expressing cells clearly indicated that following cessation of JAK3 inhibitor therapy, there appeared to be a marked increase in the NK cell lineage to preferentially express Ki67.

Taken together, the data presented herein indicate that the administration of the JAK3 inhibitor to chronically SIV infected rhesus macaques leads to a modest but consistent increase in plasma viremia and an increase in the levels of viremia in the GI tissue of these animals. This increase appeared to be associated with a loss of multiple cell lineages chief among them though being cells of the NK cell lineage. Studies are currently underway to assess the effect of JAK3 inhibitor administration during acute SIV infection in a large number of rhesus macaques, the finding of which will be published shortly. The data presented herein therefore lays the foundation for these additional studies.

## Supporting Information

Figure S1 The absolute numbers of CD3^+^. CD4^+^, CD8^+^ and CD3^−^/CD8^+^/NKG2a^+^ NK cells from each of the 6 chronically SIV infected rhesus macaques just prior to JAK3 inhibitor administration (baseline values).
(TIF)
