# Supplementary figures and images for: In Vivo Administration of a JAK3 Inhibitor to Chronically SIV Infected Rhesus Macaques Leads to NK Cell Depletion Associated with Transient Modest Increase in Viral Loads
Source: PLoS One. 2013 Jul 26;8(7):e70992. doi: 10.1371/journal.pone.0070992 (PMC3724739; doi:10.1371/journal.pone.0070992)

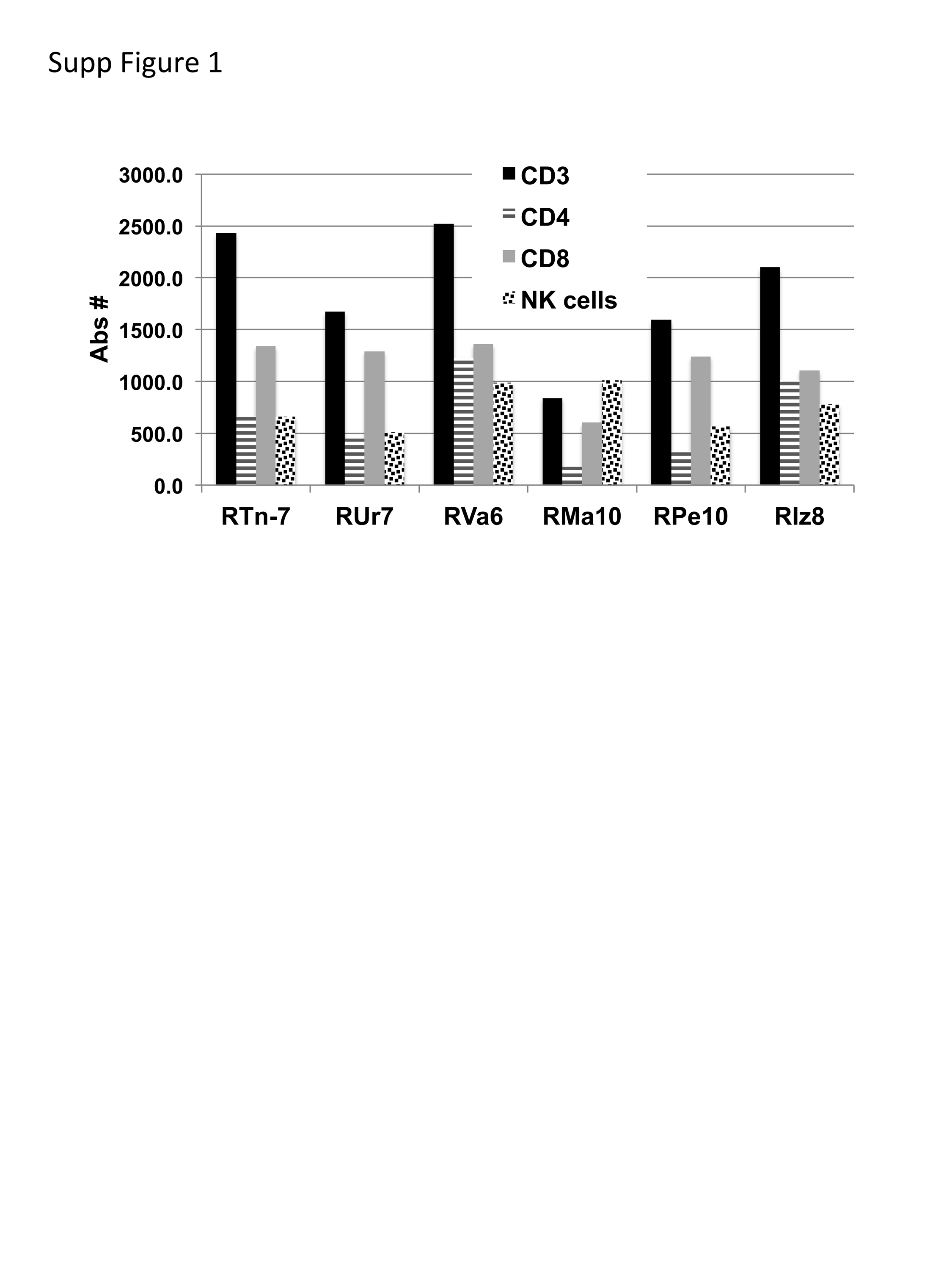

Supplement: Figure S1 — The absolute numbers of CD3+. CD4+, CD8+ and CD3−/CD8+/NKG2a+ NK cells from each of the 6 chronically SIV infected rhesus macaques just prior to JAK3 inhibitor administration (baseline values). (TIF) [file pone.0070992.s001.tif]
